# Stroke infarct volume estimation in fixed tissue: Comparison of diffusion kurtosis imaging to diffusion weighted imaging and histology in a rodent MCAO model

**DOI:** 10.1371/journal.pone.0196161

**Published:** 2018-04-26

**Authors:** Vibeke Bay, Birgitte F. Kjølby, Nina K. Iversen, Irene K. Mikkelsen, Maryam Ardalan, Jens R. Nyengaard, Sune N. Jespersen, Kim R. Drasbek, Leif Østergaard, Brian Hansen

**Affiliations:** 1 Translational Neuropsychiatry Unit, Department of Clinical Medicine, Aarhus University, Risskov, Denmark; 2 Center of Functionally Integrative Neuroscience, Department of Clinical Medicine, Aarhus University, Aarhus, Denmark; 3 Core Center for Molecular Morphology, Section for Stereology and Microscopy, Centre for Stochastic Geometry and Advanced Bioimaging, Department of Clinical Medicine, Aarhus University, Aarhus, Denmark; 4 Department of Physics and Astronomy, Aarhus University, Aarhus, Denmark; Henry Ford Health System, UNITED STATES

## Abstract

Diffusion kurtosis imaging (DKI) is a new promising MRI technique with microstructural sensitivity superior to conventional diffusion tensor (DTI) based methods. In stroke, considerable mismatch exists between the infarct lesion outline obtained from the two methods, kurtosis and diffusion tensor derived metrics. We aim to investigate if this mismatch can be examined in fixed tissue. Our investigation is based on estimates of mean diffusivity (MD) and mean (of the) kurtosis tensor (MKT) obtained using recent fast DKI methods requiring only 19 images. At 24 hours post stroke, rat brains were fixed and prepared. The infarct was clearly visible in both MD and MKT maps. The MKT lesion volume was roughly 31% larger than the MD lesion volume. Subsequent histological analysis (hematoxylin) revealed similar lesion volumes to MD. Our study shows that structural components underlying the MD/MKT mismatch can be investigated in fixed tissue and therefore allows a more direct comparison between lesion volumes from MRI and histology. Additionally, the larger MKT infarct lesion indicates that MKT do provide increased sensitivity to microstructural changes in the lesion area compared to MD.

## Introduction

Stroke is the second leading cause of death world-wide. In addition, stroke is the dominant source of adult disability and is therefore a significant health problem as well as a financial burden on society [1]. Currently there are only limited treatment options for stroke patients [2–4]. Furthermore, our ability to visualize the differential response to ischemia in stroke tissue (core, penumbra) in detail is limited although direly needed for basic research, clinical assessment and monitoring, as well as evaluation of treatment efficacy.

In the current clinical practice, diffusion weighted MRI (DWI) is often used to identify the ischemic region, which manifests as a region of decreased diffusivity compared to normal tissue. This region corresponds well to the infarct core [5, 6]. However, conventional DWI provides limited information on the microstructural changes in the ischemic penumbra [7]. Perfusion Weighted Imaging (PWI) identifies tissue with limited blood supply that is typically seen in a larger area than the DWI lesion. Thus, the DWI-PWI mismatch has been suggested to depict the penumbra [8]. While this mismatch estimates the penumbra reasonably well, it does not give an exact penumbral measure since the region of visible hypoperfusion substantially overestimates ‘true’ penumbral tissue [9, 10]. Additionally, PWI requires bolus injection, which is time consuming and prone to complications, all of which is undesirable in the acute setting. Thus, a method to identify the penumbra by increased tissue sensitivity in a fast and reliable manner, without using an external tracer, would be highly beneficial. Such methods would also be useful in preclinical studies for evaluation of potential stroke treatments by specifying the actual volume of salvable tissue.

Conventional DWI analysis assumes that diffusion weighting (*b)* is small enough to approximate water diffusion in tissue as Gaussian in all directions. This is the diffusion tensor imaging (DTI) framework. However, the microscopic composition of tissue provides numerous obstacles to diffusion (membranes, organelles etc.) causing the water diffusion profile to deviate from Gaussian as *b* is increased. Furthermore, sub-voxel domains with different diffusion properties will also contribute to this deviation [11]. Diffusion kurtosis imaging (DKI) [12] offers increased sensitivity to tissue microstructure by quantifying the deviation from free diffusion in each voxel. This is achieved by considering next term in the cumulant expansion of the signal (second order in *b*) [13]. This increases diffusion MRI’s (dMRI) sensitivity to microstructure and while indirect, the DKI technique may offer clinically valuable microstructural information not accessible with conventional DWI [14]. This could potentially enable detection of subtle changes in cellular morphology indicative of metabolically challenged tissue surrounding the infarct core. In stroke, DKI has been shown to improve detection of ischemic tissue changes compared to DTI metrics [15–19]. Nevertheless, the microstructural changes reflected by DKI-measures are not fully understood and await histological comparison, which likely needs to be not only disease specific but also take into account distinct time points in the disease progression. Such histological evaluation will ensure a correct interpretation of the DKI images and thereby potentially aid the clinical stroke setting.

The ability of DKI to detect subtle changes in tissue microstructure is well-established and has been verified by histology in a number of studies. This has e.g. been seen in a transgenic Huntington rat model [20] and in a chronic mild stress model [21], where DKI was able to detect changes in myelin protein and neurites, respectively. In experimental stroke, mean kurtosis (MK) was found to display signal intensity changes 2–3 times larger than mean diffusivity (MD) [16]. Thus, the sensitivity of DKI was found to be an advantage over DTI-based analysis, however, no infarct volume comparison or additional histology analysis were performed to elucidate the underlying morphology. In [22] *in vivo* investigations of DKI and DWI in both permanent and transient experimental stroke were performed, and here MK remained elevated even after MD had pseudo-normalized. Additionally, the authors conclude that MK is a robust and more specific indicator of microstructural alterations than MD because it is less susceptible to partial volume contamination caused by brain volume changes due to edema [22]. This indicates that MK can detect microstructural changes despite of an edema, where the more edema sensitive MD parameter will change due to the edema and not the microstructure.

The above studies provide important information on the difference between DWI and DKI, however, they compare *in vivo* MRI to *ex vivo* histology as also done in a number of other studies (e.g. [23]). This approach may create some problematic confounders. When using *in vivo* MRI to assess infarct volume, the estimate will be affected by physiological noise sources (breathing, movement etc.) as well as physiological variations in blood pressure and heart rate due to anesthesia [24] which can affect the capillary bed and thus brain tissue state. This can be additionally pronounced if longitudinal studies including multiple scan series, and thus repeated anesthesia, are performed [25]. If brains are first scanned *in vivo* and then removed for further histological analyses *ex vivo*, a confounder between the MR images and the histological images will occur as fixative alters the extracellular space [26] potentially changing the microstructural tissue composition. Therefore, direct comparison of infarct volume estimates from *in vivo* dMRI to histological estimates is error prone. Thus, it can be important to avoid these confounders and ensure the most direct comparison when investigating the morphology underlying the difference in visualization between DWI and DKI.

Sun et al. demonstrated that infarcts visible in vivo remain detectable with DWI after fixation, although with diffusivity values, but not diffusion anisotropy, altered by fixation [27]. However, DKI was not performed. In the present study, DKI is performed on ex vivo brains from a middle cerebral artery occlusion (MCAO) model of stroke followed by histology with a view on the early post-stroke phase (24 h after reperfusion). Additionally, the study utilizes a fast-DKI approach with significantly reduced DKI acquisition time. As a result, the time-scale of the scanning can be shortened to less than 1 minute [28], thereby providing cost-efficient scan times with the ability to increase scan resolution. The method can readily be translated to in vivo pre-clinical and later clinical studies, and studies have confirmed that the fast-DKI mapping is comparable to the conventional DKI, or even better due to an improved contrast-to-noise ratio (CNR) efficiency [29–31].

All in all, we here wish to explore the usage of *ex-vivo* MRI on fixed, stroke-injured rat brains as a basis for evaluation of previously reported differences in lesion volume and morphology obtained from delineation based on conventional dMRI metrics (from the DTI framework) and DKI.

## Methods

### Theory

The DKI framework hinges on a two-term expression for the logarithm of the diffusion weighted signal [12]:

$$\log S(b,\hat{n})=-bn_{i}n_{j}D_{ij}+\frac{b^{2}}{6}n_{i}n_{j}n_{k}n_{l}{\bar{D}}^{2}W_{ijkl},$$ (1)

where *b* denotes the magnitude of diffusion weighting applied along $\hat{n}=(n_{x},n_{y},n_{z})$ and subscripts label Cartesian components (e.g. *i* = *x*,*y*,*z*). Note that summation over repeated indices is indicated as $n_{i}n_{j}D_{ij}=\sum_{i,j}n_{i}n_{j}D_{ij}$ using the diffusion tensor D as an example. The kurtosis tensor W is of order four and defined in terms of spin displacement moments as in [12]:

$$W_{ijkl}=\frac{\langle R_{i}R_{j}R_{k}R_{l}\rangle -\langle R_{i}R_{j}\rangle \langle R_{k}R_{l}\rangle -\langle R_{i}R_{k}\rangle \langle R_{j}R_{l}\rangle -\langle R_{i}R_{l}\rangle \langle R_{j}R_{k}\rangle }{4{\bar{D}}^{2}\Delta^{2}},$$ (2)

where ⟨ ⟩ denotes averaging over particles, *R* = *r*(Δ) − *r*(0) is the single particle displacement, and Δ the diffusion time.

The mean (of the) kurtosis tensor (MKT or $\bar{W}$) was introduced in [28, 32]:

$$\begin{array}{l} \bar{W}=\frac{1}{4\pi }\int_{S_{2}}d\hat{n}W(\hat{n})=\frac{1}{5}\langle W,I\rangle \\ \;=\frac{1}{5}(W_{xxxx}+W_{yyyy}+W_{zzzz}+2W_{xxyy}+2W_{xxzz}+2W_{yyzz})\equiv \frac{1}{5}\mathrm{Tr}(W) \end{array}$$ (3)

where $W(\hat{n})=\hat{n}{\hat{n}}^{T}W\hat{n}{\hat{n}}^{T}=n_{i}n_{j}n_{k}n_{l}W_{ijkl}$ is the apparent kurtosis, I is the fully symmetric isotropic tensor [33], *S*_2_ is the sphere, ⟨*A*,*B*⟩ = *A_ijkl_B_ijkl_* denotes the Frobenius inner product, and Tr is the trace. From Eq (3) it is evident that six distinct $\bar{W}(\hat{n})$ terms are needed to estimate $\bar{W}$. Although not all of these can be directly estimated from a single diffusion direction (the mixed terms), all can be obtained from combinations of measurements along nine distinct directions (the three axes and two directions for each of the remaining terms to remove unwanted cross terms). Naming these directions ${\hat{n}}^{\left(i\right)},\;{\hat{n}}^{\left(i+\right)}\;\mathrm{and}\;{\hat{n}}^{\left(i-\right)}$ allows us to write [28]:

$$\bar{W}=\frac{1}{15}\left(\sum_{i=1}^{3}W({\hat{n}}^{\left(i\right)})+2\sum_{i=1}^{3}W({\hat{n}}^{\left(i+\right)})+2\sum_{i=1}^{3}W({\hat{n}}^{\left(i-\right)})\right)$$ (4)

The directions are defined as in [28] and are tabled explicitly in [34, 35]. With these directions the log-sum in Eq (4) can be constructed with Eq (1):

$$\frac{1}{15}\left(\sum_{i=1}^{3}\log S(b,{\hat{n}}^{\left(i\right)})+2\sum_{i=1}^{3}\log S(b,{\hat{n}}^{\left(i+\right)})+2\sum_{i=1}^{3}\log S(b,{\hat{n}}^{\left(i-\right)})\right)=-b\bar{D}+\frac{1}{6}\;b^{2}{\bar{D}}^{2}\bar{W}$$ (5)

Evaluated with two non-zero b-values *b*_1_ and *b*_2_, Eq (5) produces a set of two equations with $\bar{D}$ and $\bar{W}$ as the unknowns [34]:

$$\begin{array}{l} A_{1}\equiv \frac{1}{15}\left(\sum_{i=1}^{3}\log S(b_{1},{\hat{n}}^{\left(i\right)})+2\sum_{i=1}^{3}\log S(b_{1},{\hat{n}}^{\left(i+\right)})+2\sum_{i=1}^{3}\log S(b_{1},{\hat{n}}^{\left(i-\right)})\right)=-b_{1}\bar{D}+\frac{1}{6}\;b_{1}^{2}{\bar{D}}^{2}\bar{W}\\ A_{2}\equiv \frac{1}{15}\left(\sum_{i=1}^{3}\log S(b_{2},{\hat{n}}^{\left(i\right)})+2\sum_{i=1}^{3}\log S(b_{2},{\hat{n}}^{\left(i+\right)})+2\sum_{i=1}^{3}\log S(b_{2},{\hat{n}}^{\left(i-\right)})\right)=-b_{2}\bar{D}+\frac{1}{6}\;b_{2}^{2}{\bar{D}}^{2}\bar{W} \end{array}$$ (6)

With this $\bar{D}$ and $\bar{W}$ can be estimated from 19 images: one at b = 0 for signal normalization, and the nine directions at *b*_1_ and *b*_2_. For this reason we refer to the scheme as the 1-9-9 method for fast kurtosis [34] allowing robust estimation of $\bar{D}$ directly as:

$${\bar{D}}_{199}=\left(b_{1}^{2}A_{2}-b_{2}^{2}A_{1}\right)/\left(b_{1}b_{2}^{2}-b_{1}^{2}b_{2}\right)$$ (7)

and for $\bar{W}$:

$${\bar{W}}_{199}=6b_{1}b_{2}\left(A_{1}b_{2}-A_{2}b_{1}\right)\left(b_{1}-b_{2}\right)/{\left(A_{1}b_{2}^{2}-A_{2}b_{1}^{2}\right)}^{2}$$ (8)

The fast kurtosis techniques have been validated against conventional DKI in an animal model of stroke [36] and have been shown to be contrast-to-noise efficient compared to conventional DKI [30]. Clinically, fast DKI has been used to grade gliomas [37], for investigation of mild traumatic brain injury [38]. Applications also extend to imaging of the body organs e.g. renal fibrosis where optimized b-values for clinical use were provided in [35].

### The animal experiment

#### The MCAO rat model

Male Sprague Dawley rats (n = 9, 370±50 g, Taconic Europe A/S, Ry, Denmark) were housed in groups of two in a Scantainer (C-110, Scanbur, Karlslunde, Denmark) with free access to food and water in a normal 12h light/dark cycle starting at 7.00 am. The animal experiment was approved by The Danish Animal Experiments Inspectorate (ethical animal permission no. 2013-15-2934-00987) and was conducted in accordance with institutional and national guidelines for animal research provided by this agency. In the experiment, animals were anesthesized with isoflurane and euthanized with pentobarbital i.p. before perfusion fixation.

After seven days of acclimation rats were subjected to a 60-minutes middle cerebral artery occlusion (MCAO) as previously described by Belayev et al. [39] using a 4–0 reusable suture (4041PK5Re, Doccol Corporation, Sharon, USA). Rats were anesthetized with 1.8 ml/kg Hypnorm-Dormicum, intubated and mechanical ventilated at 67.0±2.0 breaths/min with an air volume of 800 cc/min (SAR-830P ventilator, Cwe Inc, Ardmore, USA). End tidal CO_2_ was continuously measured (MicroCapStar End-tidal CO_2_ analyzer, Cwe Inc., Ardmore, USA), and the breathing rate was adjusted according to relative changes in end tidal CO_2_-levels in order to maintain PCO_2_ between 35–40 mmHg. Body temperature was maintained at 37°C ensured by a feedback controlled heating pad with a rectal thermometer (Temperature control unit HB 101/2, Panlab, Harvard apparatus). The suture for MCAO was inserted via the external common carotid artery into the internal common carotid artery until the MCA was blocked. During the MCAO procedure, physiological parameters (heart rate, blood flow and systolic, diastolic and mean blood pressure) were measured by a CODA system^TM^ (Kent Scientific Corporation, Connecticut, USA) and a Laser Doppler Perfusion Monitor (Moor instruments ltd, Devon, UK) was used to confirm the blockage of the artery. Post-surgically, the animals were housed separately to ensure a calm awakening, avoid wound biting by cage mates and to ease the monitoring. Additionally, Temgesic (0.03 mg/kg, RB Pharmaceuticals limited, Berkshire, UK) was administered sc. as analgesic to avoid any unnecessary pain. The animals were checked every 30 minutes for the first 2 hours post-surgery and from then on at regular, longer intervals if the animal was pain-free and stable. If any animal displayed severe discomfort and pain indicated by immobility, vocalization and breathing difficulties, the animal was euthanized immediately.

#### Brain fixation

After 24 hours of reperfusion the animals were deeply anaesthetized with pentobarbital (1 ml) and perfused intracardially using a 10 IE/ml heparin-saline solution followed by 4% formaldehyde (10% formalin, buffered solution, pH 7,0 ±0,2, VWR chemicals, Leuven, Belgium). The brains were subsequently removed from the skull and stored in 4% formaldehyde.

### *Ex vivo* MRI

#### Sample preparation for preclinical MRI

To improve the MRI signal, the brains were rinsed in PBS 24 hours before imaging to remove the formaldehyde-solution. Brains were compactly packed into tubes containing Fluorinert (FC-770, 3M, St. Paul, MN, USA) prior to MRI.

#### Diffusion MRI protocol

MRI was performed on a Bruker Biospec 9.4 T animal system with Bruker BGA12S-HP gradients (660 mT/m) using a 76 mm quadrature volume coil (Bruker Biospin, Ettlingen, Germany). Anatomical scans were performed with a RARE sequence with the following parameters; Echo time (TE) of 20 ms, RARE factor 8, 16 averages and a repetition time (TR) of 15204.8 ms. The slice thickness was 0.25 mm, in-plane resolution was 0.125 x 0.125 mm^2^ and the total number of slices was 80. Scan time for the anatomical data acquisition was 2 hrs 38 min.

Diffusion data was acquired with the 1-9-9 method outlined above and in [28]: 1 image with b = 0 ms/μm^2^, 9 images with b = 1 ms/μm^2^ and 9 images with b = 2.5 ms/μm^2^. The dMRI data was acquired with the following imaging parameters: TE = 27.387 ms, TR = 7500 ms, and diffusion timings δ/Δ = 6/14 ms. Data was acquired with an in-plane resolution of 0.25 x 0.25 mm^2^, and a slice thickness of 0.5 mm. Whole brain data was acquired (30 slices in total) with two averages. Scan time for the DKI protocol was 14 hours.

#### MRI data analyses

All dMRI data analysis was performed in Matlab (Matlab R2015b, The Mathworks, Natick, MA, USA). From the 1-9-9 data mean diffusivity (MD) and (MKT, $\bar{W}$) were estimated using Eqs (7) and (8) respectively. The infarcted area was outlined manually on maps of MD and MKT by a single blinded researcher by adding overlay masks to each MR image. The masks were added by first selecting a region of interest (ROI) and then precisely adjusting the mask one voxel at a time. From these, total infarct volume estimates were obtained based on MD and MKT separately. For each brain, whole brain volume was also estimated based on the MD images. The MRI infarct and hemisphere volumes were calculated by the amount of voxels within the designated masks multiplied by the volume of each voxel.

### Histology

After MRI, the perfusion-fixed brains were placed in 30% sucrose solution for 48 hours. The brains were covered with Tissue-Tek O.C.T. compound (Sakura Finetek, Torrence, CA, USA.) and frozen on the fast-freeze area in a cryostat (-56 ^o^C, 20 minutes, Microm HM560, Thermo Scientific, Waltham, USA) before being cut into 40 μm coronal sections (Knife and sample temperature -18 ^o^C, Microm HM560, Thermo Scientific, Waltham, USA). Based on a systematic sampling principle, a sampling fraction of 1/15 sections were selected and stored in De Olmers solution.

#### Histological staining

The sections were mounted on non-pre-coated glass slides (Superfrost, 76 x 26 mm, cut edges, Thermo Scientific, Waltham, USA) and stained with hematoxylin (H-stained; 0.1%, Hematoxylin Mayer Sur, The pharmacy of the Capital Regional Hospital, Denmark) for 20 minutes followed by a dehydration procedure.

#### Volume measurement on H-stained brain sections

The infarcted and individual hemisphere areas were estimated by a single blinded researcher using the Cavalieri estimator [40] in the NewCAST software (Visiopharm Integrator System, version 5.3.1.1540, Visiopharm, Hoersholm, Denmark). The sections were visualized by an Olympus MacroView microscope (Olympus MVX10, Olympus, Denmark) containing a 0.63X lens (Olympus MVPLAPO) with a digital camera (Olympus DP71, Olympus, Denmark). The individual hemispheres were summed to give whole brain areas. The total infarct volumes were estimated by the following formula:

$$V=t∙\frac{1}{SSF}∙\sum a_{i}$$ (9)

Where t is the section thickness (40 μm), *SSF* is the section sampling fraction (1/15) and *a*_*i*_ is the cross sectional area for each section. The cross sectional area (a_i_) was calculated using a 2D nucleator [41].

### Statistics

Two of the primary brains showed no infarct volume on the hematoxylin stain due to preparation issues and these data points were thus removed from the analysis. Since histology was used as the standard for comparison, the corresponding data points for MD and MKT were also removed.

GraphPad Prism (version 7.0, Graphpad Software, La Jolla, USA) was used to analyze all data. The data was checked for a normal distribution using the Shapiro-Wilk normality test. All data was normally distributed. The brain hemispheres and infarct volumes were tested by a paired parametric t-test. Data was considered significant at the α≤0.05 level. All data are showed as mean+/-SD. Intraclass Correlation Coefficient (ICC) using a two-way mixed effects model (absolute agreement) were used to assess both the intra- and inter-variability using the SPSS statistical package version 22 (SPSS Inc, Chicago, USA).

## Results

### Whole brain volumes

**Fig 1:**
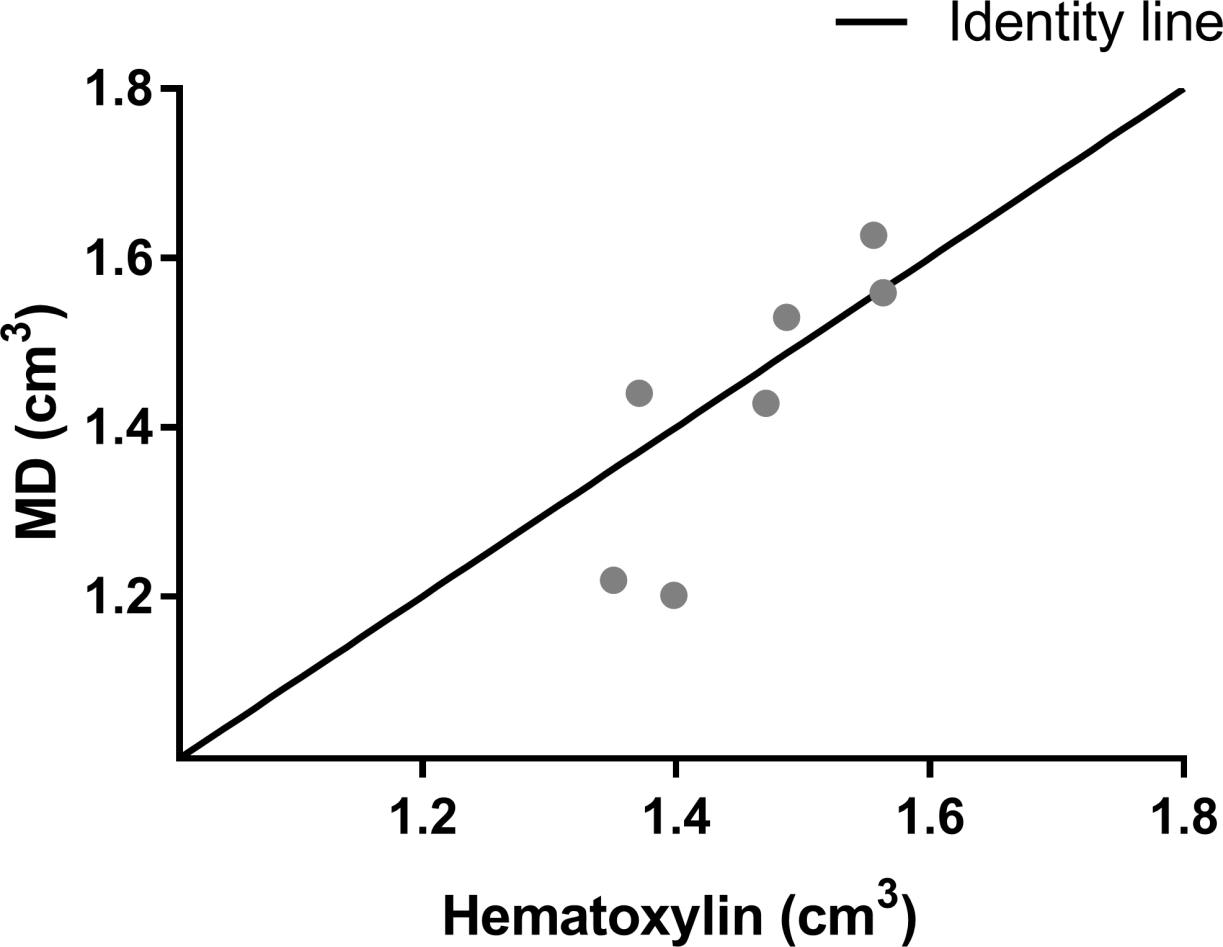
Whole brain volumes. The whole brain volumes measured by MD and hematoxylin stain. (n = 7). Data are represented as a scatter plot. MD: Mean diffusivity.

The whole brain volumes for MRI and hematoxylin were measured to assess if the histological processing (freezing) of the brains had changed the overall brain volumes which would make a direct comparison between methods unreliable. The whole brain volume measured by both MD (1.429 cm^3^+/-0.1646) and hematoxylin (1.457 cm^3^+/-0.08595) did not differ significantly between measurement techniques (p = 0.5106, df = 6) (Fig 1) (Table A in S1 File).

Since the brains had a constant volume during infarct volume measurements by both MRI sequences and the whole brain volume is similar for MD and hematoxylin, then an accurate comparison between all infarct volumes is assumed.

### Infarct volumes

**Fig 2:**
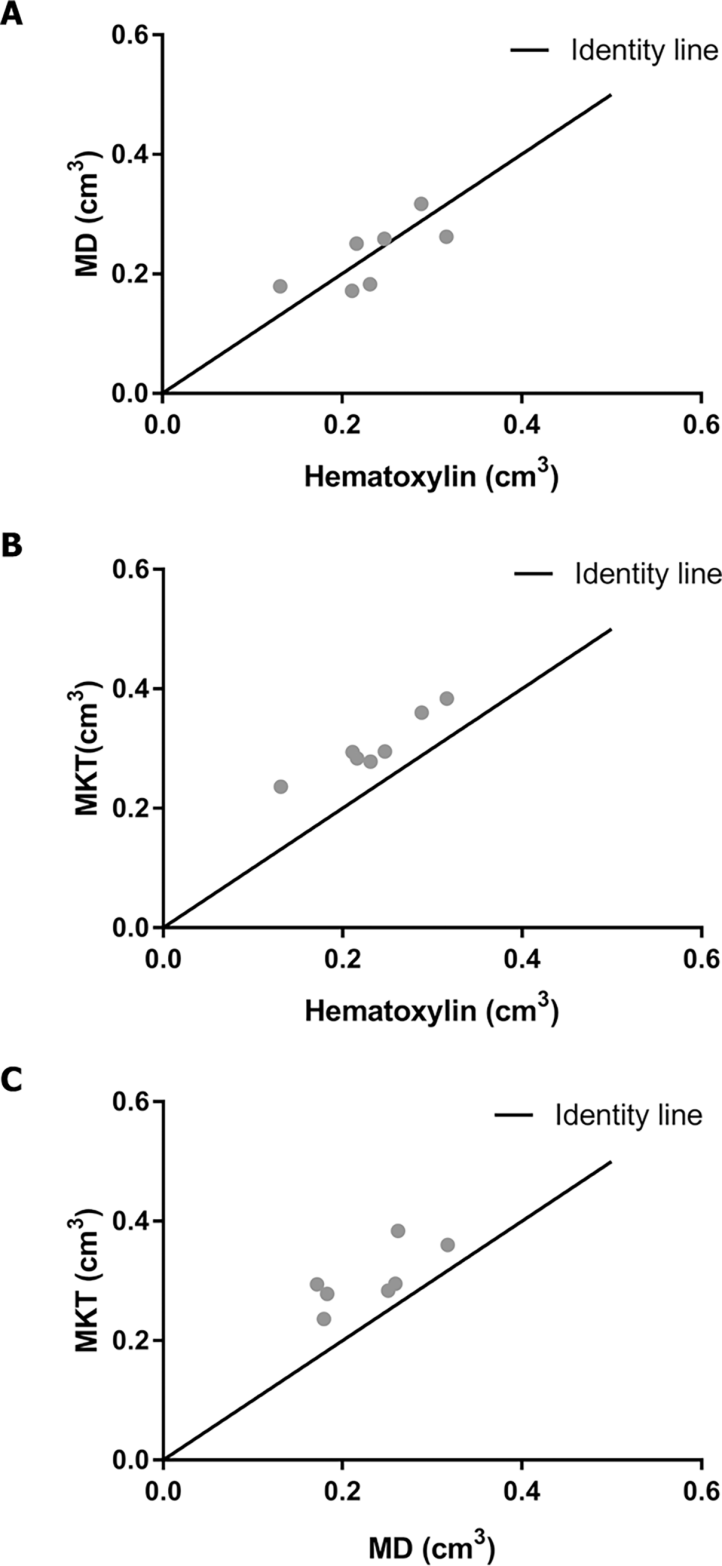
Infarct volumes. The infarct volumes visualized as the correlation between A; MD and Hematoxylin, B; MKT and Hematoxylin and C; MKT and MD (n = 7). MD: Mean diffusivity. MK: Mean Kurtosis Tensor.

The infarct volumes were measured directly for all three imaging methods (Table B in S1 File). The infarct volumes did not differ significantly between MD (0.232 cm^3^+/-0.021) and hematoxylin (0.234 cm^3^ +/-0.023) (p = 0.8825, df = 6) (Fig 2A), however the infarct volumes measured by MKT (0.304 cm^3^ +/-0.019) were larger than both hematoxylin (p<0.0001, df = 6) (Fig 2B) and MD (p = 0.0028, df = 6) (Fig 2C).

**Fig 3:**
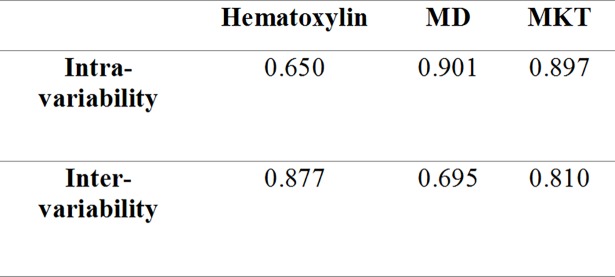
The intra- and inter-variability. The intra- and inter-variability obtained from all three infarct visualization methods. MD: Mean diffusivity. MK: Mean Kurtosis Tensor.

The intra- and inter-variability were measured on three randomly selected brains to assess the reliability of the obtained results. The primary assessor and a second designated assessor re-estimated the infarct volumes for all three visualization methods (Table C in S1 File). The variability indicated by ICC is depicted in Fig 3. Additionally, one-way ANOVA was used to assess the data points for the three brains across all visualization methods and no significant differences were found for assessment by the primary assessor or between assessors.

**Fig 4:**
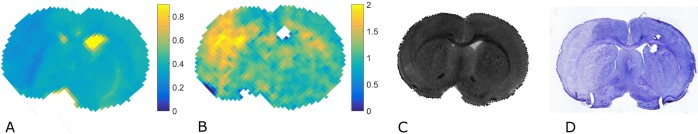
Example of difference in visualization. Infarcted area depicted by A) MD B) MKT C) Anatomical RARE image and D) Hematoxylin stain to illustrate the difference in resolution and contrast. MD: Mean diffusivity. MKT: Mean Kurtosis Tensor.

In all, MKT indicates changes in a larger volume than can be detected by either hematoxylin or MD. An example of the three different imaging methods is provided in Fig 4.

### Post-hoc analysis

**Fig 5:**
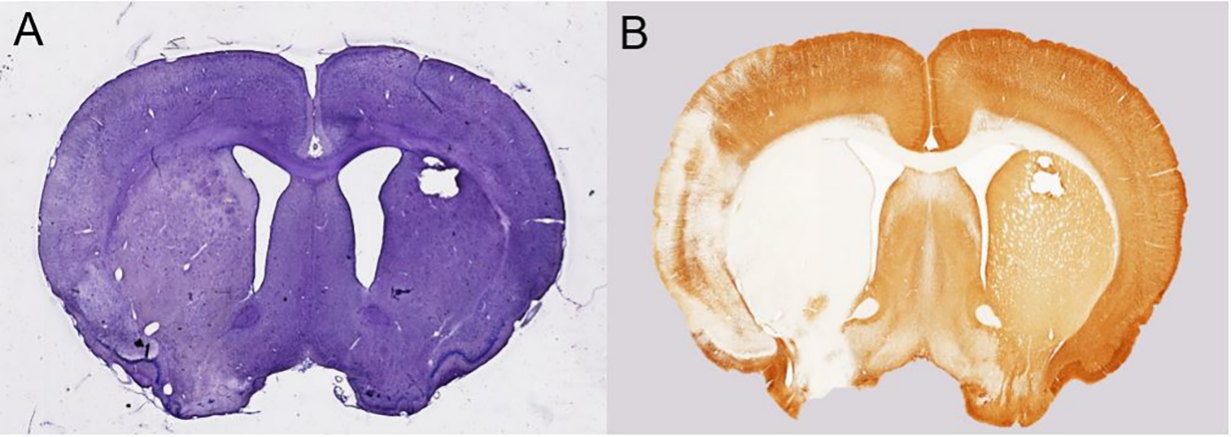
Example of the H- and MAP2-stained post-hoc brains. Infarcted area on an additional post-hoc brain depicted by A) Hematoxylin stain B) MAP2-stain.

Two additional MCAO brains (same animal protocol) were stained with hematoxylin and anti-Microtubule-Associated Protein 2 (MAP2) antibody. Both post-hoc brains depicted a larger infarct volume for the MAP2 stain (1^st^: 0.181 cm^3^, 2^nd^: 0.184 cm^3^) compared to the hematoxylin stain (1^st^: 0.162 cm^3^, 2^nd^: 0.117 cm^3^). An example of the two staining methods is provided in Fig 5.

## Discussion

The present study shows that the infarcted area remains MR-visible post-fixation even at this early time-point. Disparate infarct volume estimates were obtained from mean diffusivity ($\bar{D}$ or MD) and mean kurtosis tensor ($\bar{W}$ or MKT). Additionally, the infarct volume estimates based on MD agree with hematoxylin stained infarct volume estimates whereas the MKT estimates a significantly larger infarct. The use of perfused-fixed brains for the entire study made it possible to compare the MRI results and the results from histological investigation directly. Additionally, the usage of *ex vivo* brains and the new fast-DKI sequence can notably increase the obtained image resolution. This can facilitate further morphological investigations.

The study has a large comparative power since all infarct analyses are performed *ex vivo*, consequently providing fewer confounds. By using an *ex vivo* scanning method the study avoids contamination from physiological sources of noise (pulsation, respiration, swallowing etc.) during scanning and is furthermore not affected by the morphological changes that occur during fixation of the brain [26]. Consequently, the tissue state at the time of scanning and histology is more directly comparable. One can certainly argue that the findings obtained from scanning of fixed ex-vivo tissue cannot be directly translated to the in-vivo stroke setting, however it can create pre-clinical knowledge that onwards could aid clinical investigations.

The infarct volumes measured by MKT differ from both MD and hematoxylin. It is a general notion that DWI largely represents the ischemic core [5, 6]. Thus, we speculate if the larger infarct volume measured by MKT corresponds to a measurement of the core as well as a metabolic-challenged tissue surrounding the core.

In [30], the detected MD infarct is larger than the MKT infarct after 1 hour of ischemia. However, they perform a permanent occlusion and therefore have no reperfusion injury, which might explain why the MD infarct is larger than the MKT infarct at this early time point. In addition, Hui et al. [22] state that MD renormalizes 1–2 days after ischemia while the MK remains elevated for up to 7 days. The difference in infarct volumes could potentially to some extent be due to this normalization. Additionally, Wu et al. [30] performed their study *in-vivo* where our *ex-vivo* scan requires a fixation prior to the scan which will decrease the water content of brain and thus affect the MD more than the MKT parameter.

The brains were fixed 24 hours post stroke, and even though the majority of the penumbra is generally consumed by the core 6 hours post stroke, a small penumbra fraction can persist up to 48 hours post stroke [7, 42, 43]. This indicates that our DKI sequence could potentially detect a remaining penumbra or at least a metabolic-challenged tissue area.

A limitation of using manual mapping for infarct estimation is the subjectivity in rating. This limitation is addressed by using blinded assessors. In this study the primary assessor was blinded, however the clear distinction between the MD, MKT and histology sections did create the potential for bias of the infarct estimation. To ensure the best possible infarct volume estimations, the primary assessor was trained by an expert neuroradiologist for assessment of the MRI images and was already familiar with histological infarct estimation.

An automated infarct estimation technique for all infarct visualization methods would surely solve the potential bias. Although several automated methods are known for MD [44] and a new method has even been proposed for assessment of kurtosis images [45], automated infarct estimation for histology is, to the best of the authors’ knowledge, currently not possible. Thus, to ensure a comparable infarct estimation across all methods, manual assessment was deemed the most reliable method. If the MR images were automatically assessed and then compared to manually assessed histology sections, a larger risk of bias would occur than having the same manual assessment across all methods. Additionally, the analysis of intra- and inter-variability indicates that we have used robust methods with good reliability of our results.

In patients with stroke, brain edema develops over the first 24–48 h and reaches its maximum between 3–5 days [46]. However, in rat MCAO experiments, the edema is largest at an earlier time point ranging from 24 h [47] to 72 h [48]. Consequently, the current study investigates the morphology at a time point where edema volume could potentially peak and it is therefore a parameter to consider. To estimate the infarct volumes, we employed a direct measurement method for all visualization techniques. However, when assessing the individual hemispheres, the left hemisphere volume was larger than the right hemisphere for both histology (8.9%) and MD (4.4%). Since the individual hemisphere volumes did differ, it was necessary to consider using edema correction for the infarct volumes (formula proposed by Swanson et al. [49]). This correction ensures that edema does not have an effect on the infarct volumes. Nonetheless, the usage of the direct method was agreed upon based on several factors: The current study does not evaluate a new treatment aspect, where a precise assessment of infarct volume reduction is required. Additionally, edema will also be present in *in vivo* scans and thus it is interesting to asses MD and MKT parameters’ ability to detect an infarct area affected by edema.

In the ischemic brain at 24 hours post stroke, cellular swelling of astrocytes and shrinkage of neurons are visible [50] along with initial axonal swelling and large hemispheric swelling [51]. In the study by Li et al. [52], three different zones (ischemic core, inner and outer boundary) are defined according to the cell viability and morphological changes. In the ischemic core, 24.7% of all cells have disappeared and 55.2% of all cells are irreversibly damaged, 49.9% of which are necrotic cells. The amount of viable cells is larger in the inner and outer boundary, and the tissue structure is more maintained, with only 15.9% and 9% disappeared cells, respectively. As the tissue structure is affected by different stroke etiology, occlusion and reperfusion times, this hampers a uniform description of the core and penumbra. Yet, the cellular changes that commonly exist (necrosis, disappeared cells and changed cell morphology) should be detectable by kurtosis, since the sequence has shown increased sensitivity to microstructure [12]. Therefore, kurtosis does potentially have the ability to distinguish between the core and penumbra by novel microstructural information and can possibly aid both researchers and clinicians in the future of stroke treatment.

For the additional investigation of penumbra detection by kurtosis, a display of the H-stained sections as an overlay on the MR images would have been optimal. The MRI mismatch could then be analyzed in direct comparison with histology and compared to the findings by Li et al. [52]. This would provide histological information on the mismatch area and thus potentially confirm or dismiss our hypothesis of kurtosis’ ability to distinct between core and penumbra. However, this was not possible in the current experimental setup since the histological sections had not been perfectly matched anatomically with the MR images. Thus, a comparison of the MRI mismatch area to the histological sections would not be reliable and instead pose a risk of drawing conclusions in error. Overall, the study is a preliminary study that hopes to inspire future work where the possibility for overlay would be included in the study design.

In the recent study by Rudrapatna et al. [17], MRI and immunohistochemistry (IHC) of fixed rat stroke brains were performed eight weeks post stroke. Unlike the current study, the authors did not evaluate the difference in infarct volume but rather differences in MRI parameters compared to different IHC “amount of contrast” measures. The authors report considerable DTI/DKI mismatch and find that perilesional areas depicting glial, inflammatory and neuronal changes detected by IHC also displayed larger changes for MK than for MD. However, it was impossible to directly associate the MRI parameters to morphological features since the study found inconsistencies across the contralateral and ipsilateral hemispheres. In all, this study support our assumption that MKT could detect microstructural changes, but also highlights the need for more information.

The post-hoc analysis with MAP2 staining was performed to shed some light on the above matter. MAP2 is a key element in the neuronal cytoskeleton by regulating microtubule stability and thus overall dendrite structure [53]. In the ischemic brain, MAP2 identifies ischemic damage indicated by dendrite cytoskeletal degradation, where the degree of degradation correlates with the attenuation of immunoreactivity [54]. Dendrites are susceptible to ischemic stress [55, 56], and consequently dendrite damage could occur in absence of neuronal death making the neuronal damage at these brain areas reversible. A macroscopic investigation of the MAP2-stained sections revealed a larger infarct size compared to the H-sections with an attenuation of MAP2 staining in the periphery and a total loss of staining in the center of the damaged area. One might therefore speculate that in our study MKT detects subtle forms of neuronal damage (dendrite atrophy) at both reversible and irreversible stages, whereas MD only reveals areas with a total or relative large percentwise loss of dendrites.

An ischemic stroke confers many structural cell changes, so the actual cellular composition that provides the MKT value might vary during stroke progression and thus signify the importance of image acquisition time after stroke. The fixed tissue imaging method used in this experiment could yield more information about the relation between dMRI metrics and histological lesion composition. This could potentiate more controlled longitudinal studies, where changes in infarcted tissue and cellular composition over time could be elucidated by harvesting brains at multiple time-points post stroke. This might reveal which morphological changes can be detected by either MD or MKT in the acute and sub-acute phases post stroke. The fixation also enables longer scanning sequences, and thus the maximum resolution is determined by the MRI scanner hardware and not by the limitations imposed by the experimental challenges of in-vivo scans. The increased scan time can be outweighed by the ability to scan several brains in one session, thereby making it more cost-efficient.

Altogether, the present study supports that MKT could in fact provide a different structural knowledge undetectable in a regular MD measurement. Additionally, we demonstrate a valuable method for comparison of stroke infarct volumes in fixed tissue across methods. This can aid the pre-clinical research in obtaining histological validation of the DKI parameter. Previously, the kurtosis sequence could not be used in the clinic due to the long scan time, however the new fast-kurtosis sequence has made this possible. Hence, the MKT measure could in a future clinical setting create the basis for a DWI-DKI-mismatch analysis, specifying the actual volume of salvable tissue or revealing treatment effect on damaged microstructure.

## Supporting information

S1 File **Table A.** Data of the whole brain volumes. **Table B.** Data of the infarct volumes. **Table C.** Data of the re-analyzed infarct volumes.
(XLSX)
